# The complete plastid genome sequence of garlic *Allium sativum* L

**DOI:** 10.1080/23802359.2016.1247669

**Published:** 2016-11-12

**Authors:** Mikhail A. Filyushin, Alexey V. Beletsky, Alexander M. Mazur, Elena Z. Kochieva

**Affiliations:** a Institute of Bioengineering, Research Center of Biotechnology of the Russian Academy of Sciences, Moscow, Russia;; b Faculty of Biology, Lomonosov Moscow State University, Moscow, Russia

**Keywords:** Garlic, chloroplast genome, next-generation sequencing, *Allium sativum*

## Abstract

The complete plastid genome sequence of garlic *Allium sativum* was determined using Illumina sequencing. The plastid DNA is 153,172 bp in length and includes a large single copy region (LSC) of 82,035 bp and a small single copy region (SSC) of 18,015 bp, which are separated by a pair of 26,561 bp inverted repeat regions (IRs). In total, 134 genes are identified, containing 82 protein-coding genes, 38 tRNA genes, eight rRNA genes and six pseudogenes. Most of genes occur as a single copy, while 19 genes are duplicated in IRs. Among 15 intron-containing genes, *clpP* and *ycf3* contain two introns and the rest have one intron.

Garlic (*Allium sativum* L.) is the second most important crop of the genus *Allium* after the bulb onion. It is cultivated and consumed worldwide and is popular for its nutritional and medicinal properties. Garlic production worldwide is estimated at more than 24 million tons and is steadily growing. Garlic cultivars are sterile and thus propagate only asexually. It was proposed that garlic originated in Central Asia and due to high ecological plasticity as well as to active trading, has spread throughout the world (Vavilov 1951; Hong & Etoh 1996).

*Allium sativum* is a monocotyledonous plant and belongs to section *Allium* genus *Allium* (family Amaryllidaceae order Asparagales), which contains more than 750 species (Friesen et al. 2006).

For sequencing *A. sativum* accession from Uzbekistan was chosen (specimen voucher VSRI: 31, Vavilov All-Russian Scientific Research Institute of Plant Industry). The complete garlic plastid genome was estimated by the high-throughput sequencing on the Illumina HiSeq 1500 Sequencing System (Illumina, CA). The plastid genome was assembled with SPAdes v3.8 (Bankevich et al. 2012) and manually finished with additional sequencing and *Allium cepa* (KF728079) as the reference. The resultant plastid genome was annotated by using the DOGMA program (http://dogma.ccbb.utexas.edu) (Wyman et al. 2004) and by comparing with those of *A.  cepa* (KF728079, KF728080, KM088013, KM088014) (von Kohn et al. 2013; Kim et al. 2015). A physical map of the *A. sativum* plastid genome was generated using the web tool OGDRAW (http://ogdraw.mpimp-golm.mpg.de) (Lohse et al. 2013). The complete plastid genome sequence was submitted to GenBank with accession number KX683282.

The garlic plastid genome is 153,172 bp in length and comprises a large single copy region (LSC, 82,035bp), small single copy region (SSC, 18,015 bp) and two inverted repeat regions (IRs, 26,561bp).

**Figure 1.**
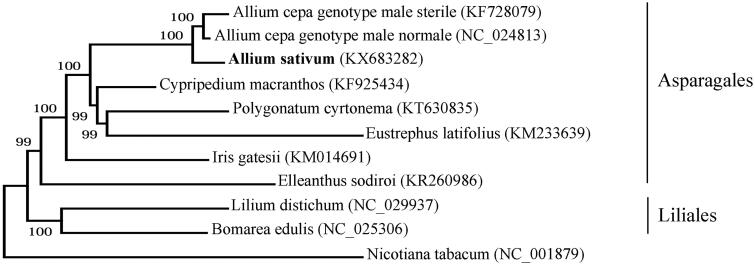
Phylogenetic tree inferred by maximum-likelihood using 82 protein-coding gene sequences of 10 species including seven species from the Asparagales order: *Allium cepa* (genotype male sterile KF728079 and genotype male fertile NC_024813), *Allium sativum* (KX683282), *Eustrephus latifolius* (KM233639), *Polygonatum cyrtonema* (KT630835), *Cypripedium macranthos* (KF925434), *Elleanthus sodiroi* (KR260986), *Iris gatesii* (KM014691); two species from Liliales order: *Bomarea edulis* (NC_025306), *Lilium distichum* (NC_029937); and *Nicotiana tabacum* (NC_001879) as an outgroup. PhyML 3.1 (Guindon et al. 2010) was used for the sequence alignment and construction of the tree. Bootstrap support values based on 1000 replicates are displayed on each node.

The plastid genome harbors 134 genes that include 82 protein-coding genes, 38 tRNA genes, eight rRNA genes and six pseudogenes. Most of them are single copy genes, whereas 19 genes present in double copies, including six protein-coding genes (*rps19*, *rpl2*, *rpl23*, *ycf2*, *ndhB*, *rps7*), nine tRNA genes (*trnR*-ACG, *trnM*-CAU, *trnL*-CAA, *trnV*-GAC, *trnH*-GUG, *trnI*-CAU, *trnI*-GAU, *trnA*-UGC, *trnN*-GUU) and all four rRNA genes in IRs (*rrn4.5*, *rrn5*, *rrn16* and *rrn23*). Intron sequences are found in 15 genes, 13 (*atpF*, *rpoC1*, *trnL*-UAA, *trnV*-UAC, *ndhA*; four genes in IRs: *rpl2*, *ndhB*, *trnI*-GAU, *trnA*-UGC) of which contain a single intron while two (*clpP* and *ycf3*) have two introns. Six genes became pseudogenes due to internal stop codons identified in their coding sequences (*rps2*, *rps16*, *infA*, two *ycf15* in IRs) or because of incomplete duplication in the IRB/SSC junction region (*ycf1*).

Sequence comparison of *A. sativum* and *A. cepa* plastid genomes reveals similar gene order (von Kohn et al. 2013; Kim et al. 2015). Compared to *A. cepa*, in the plastid genome of *A. sativum* seven deletions (18–221 bp) in intergenic spacers and a number of short insertions (2–31 bp) are identified.

Phylogenetic analysis inferred from 82 protein-coding genes of plastid genome showed a close relationship of *A. sativum* and *A. cepa* (Figure 1).
